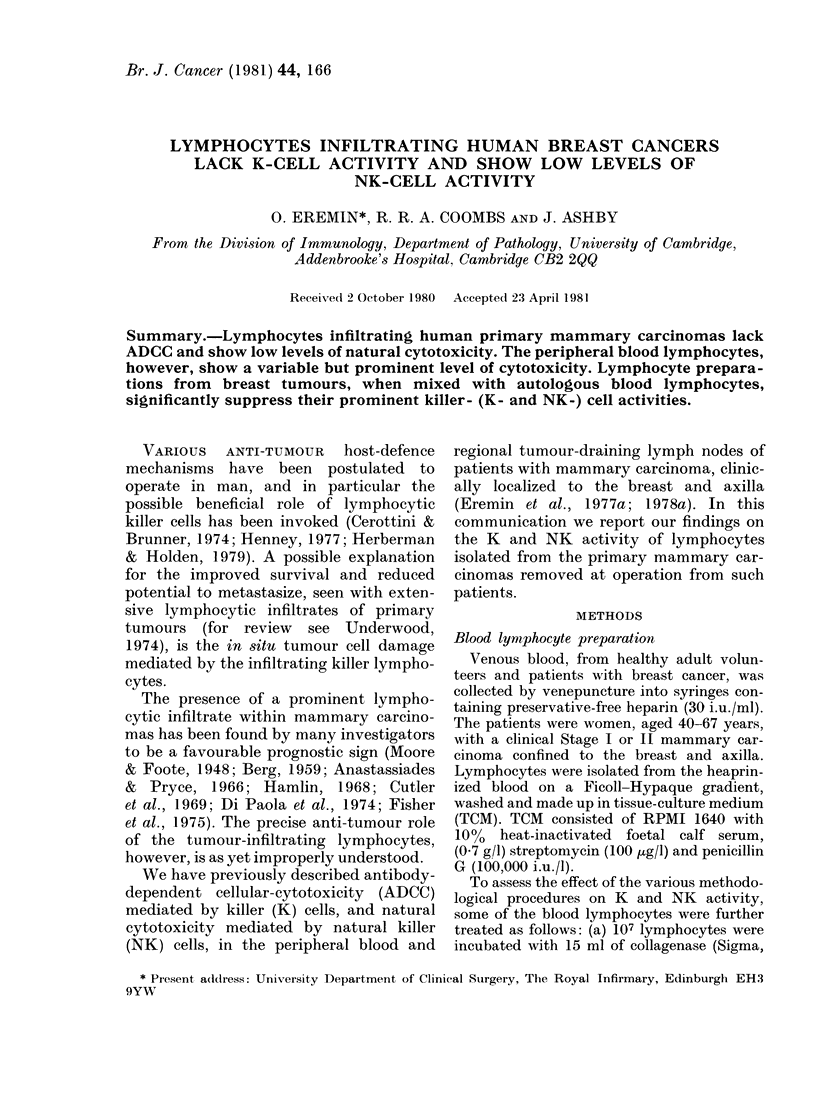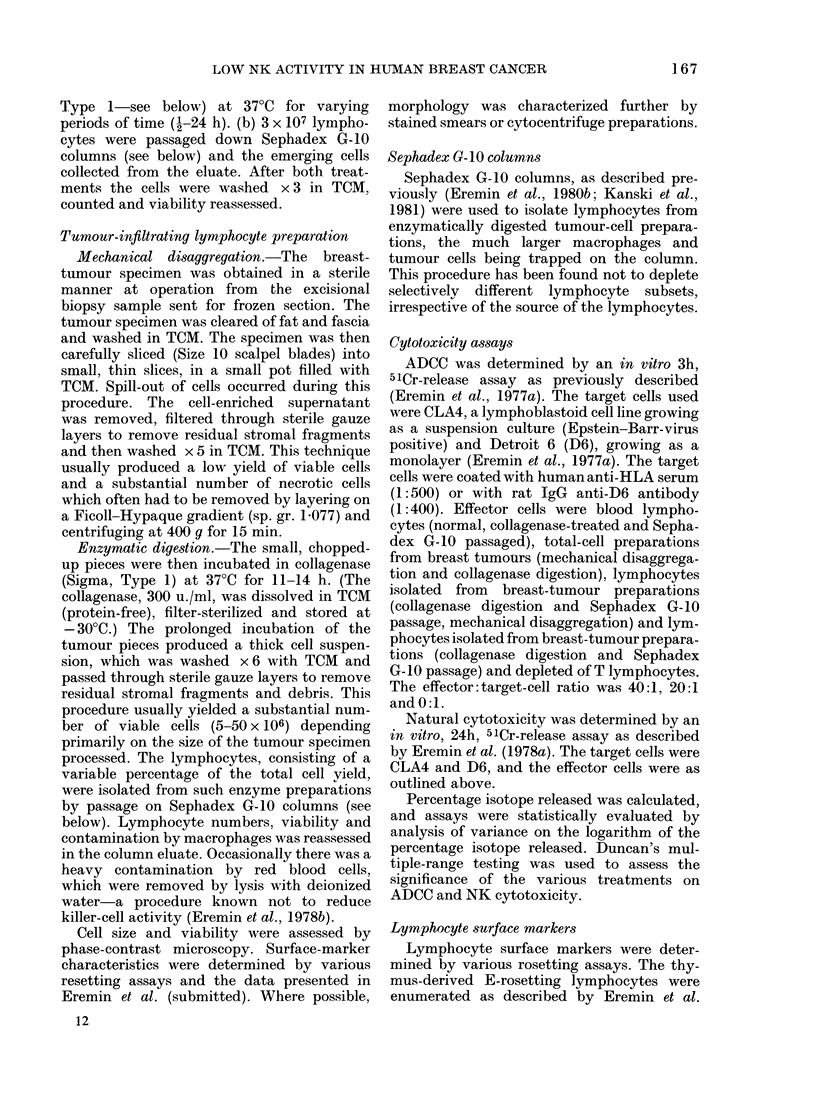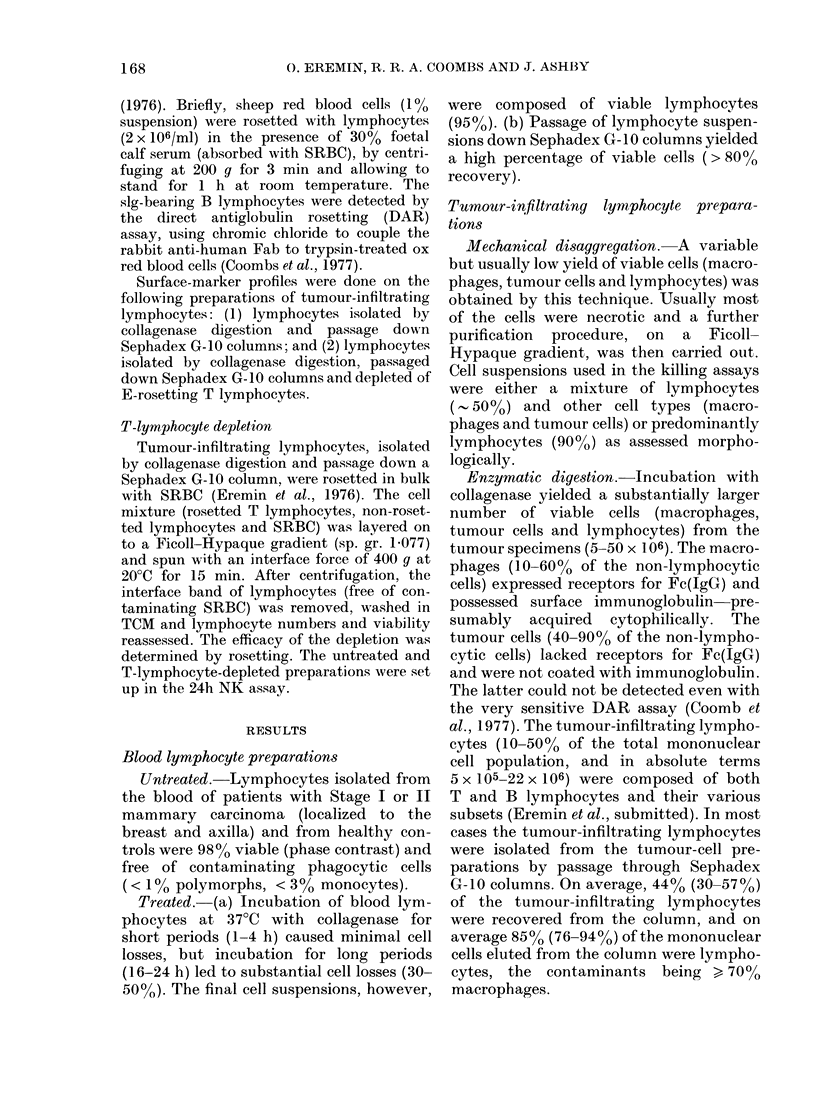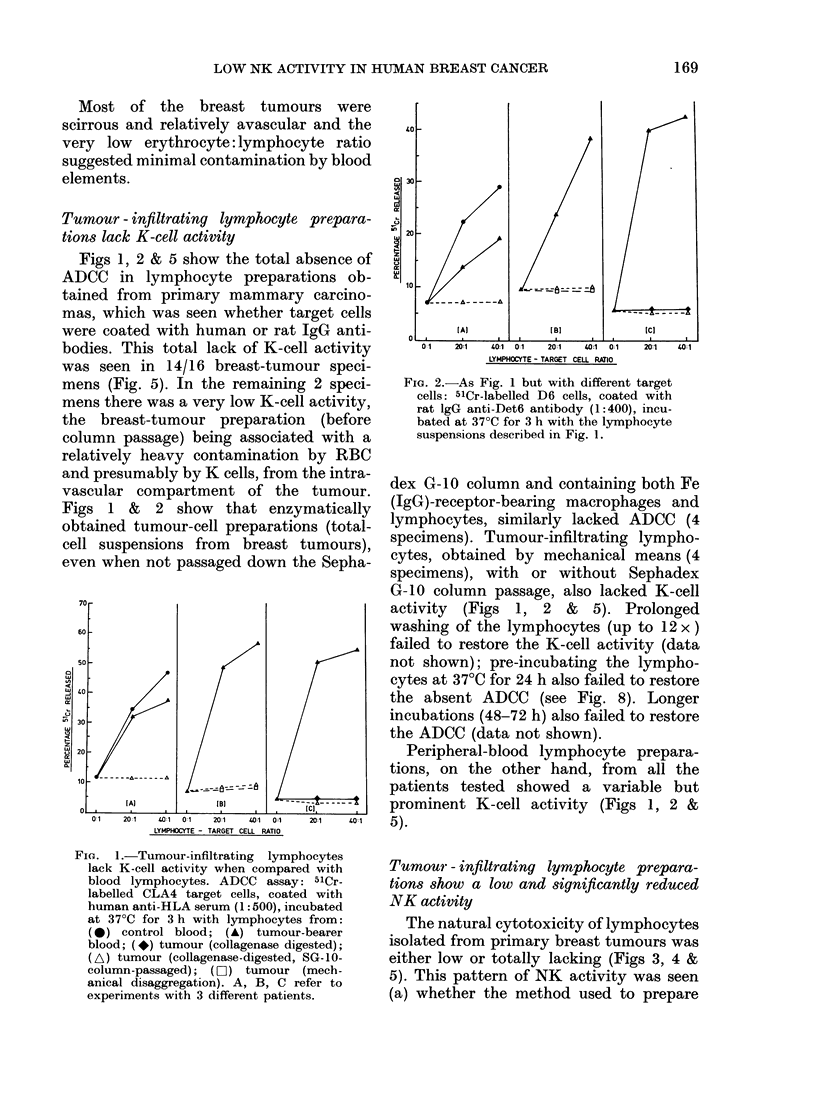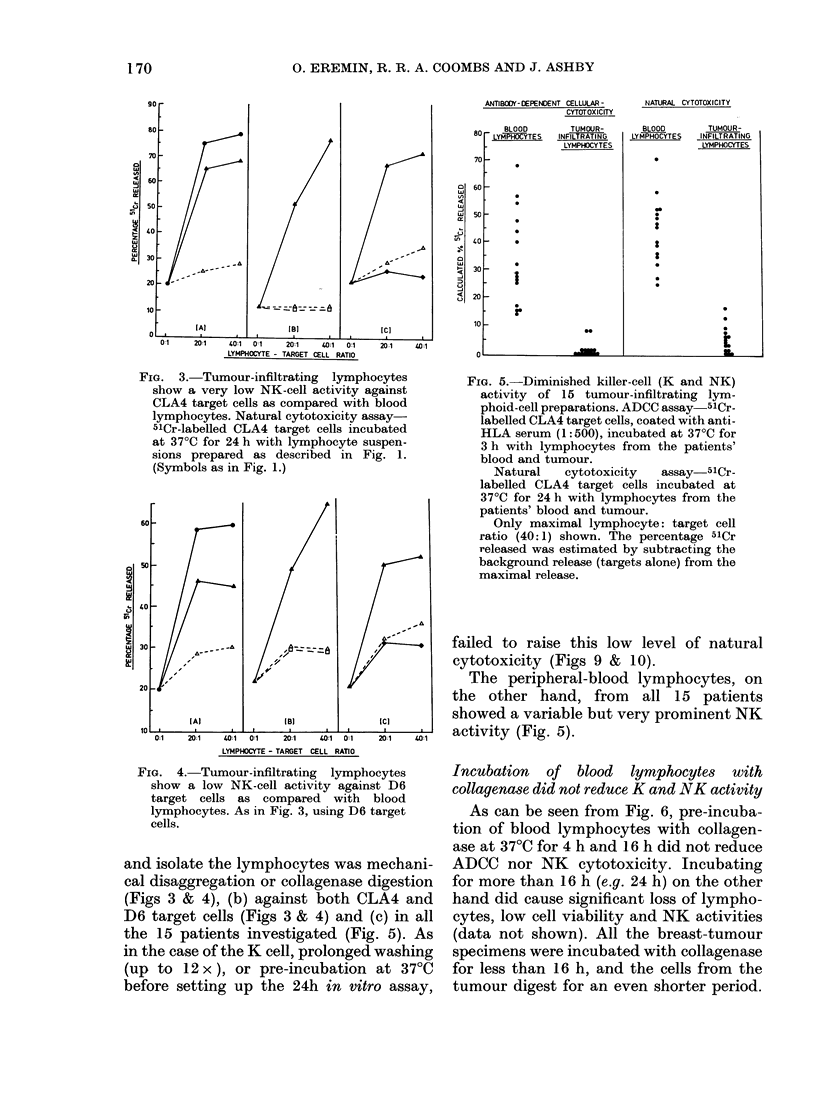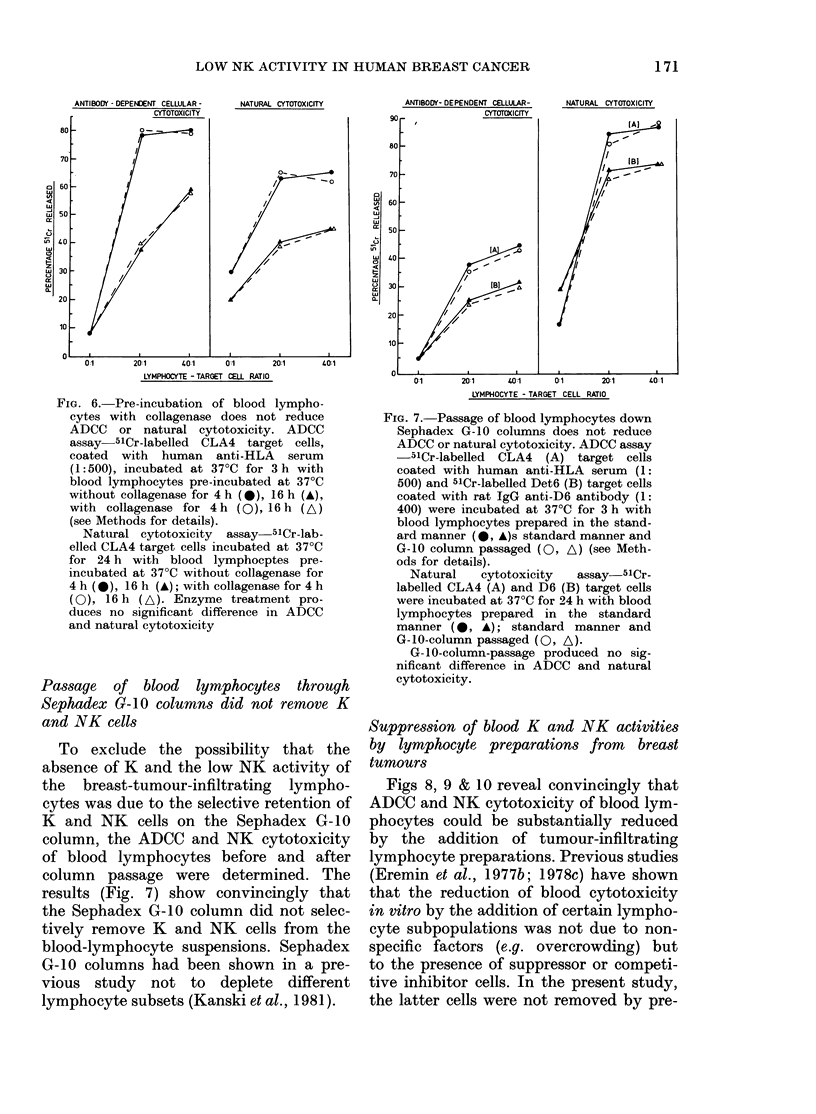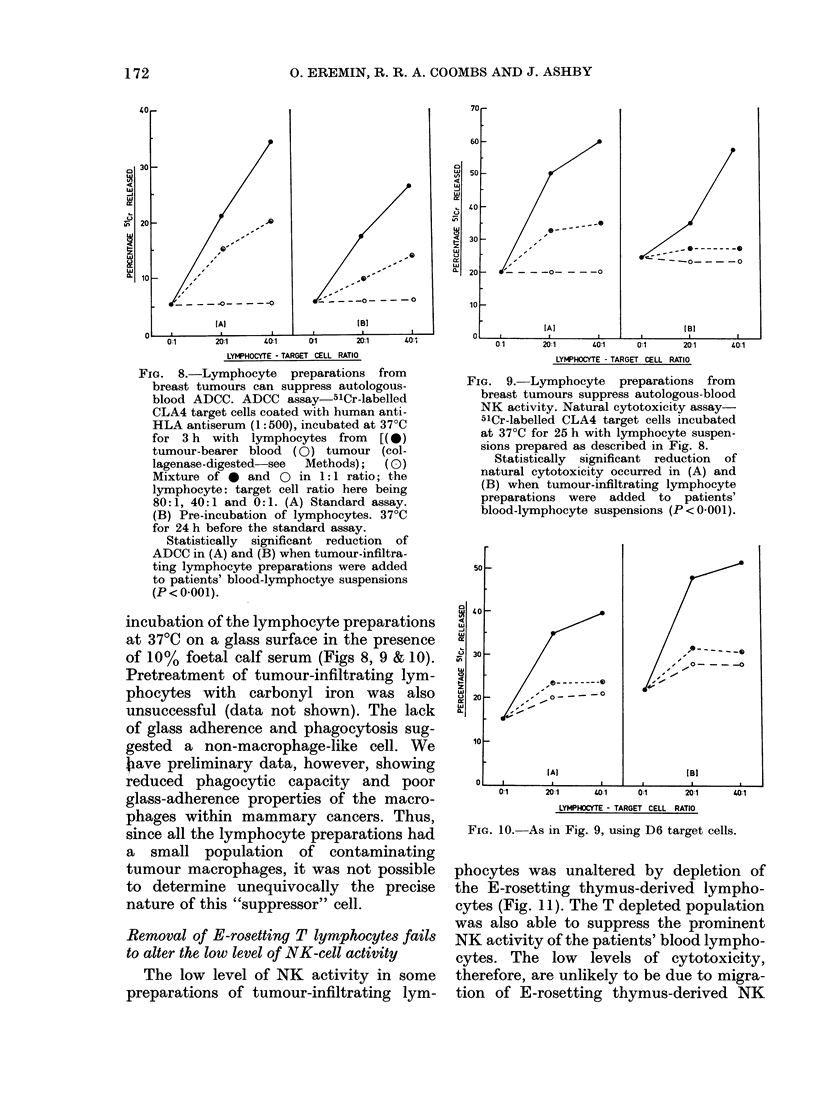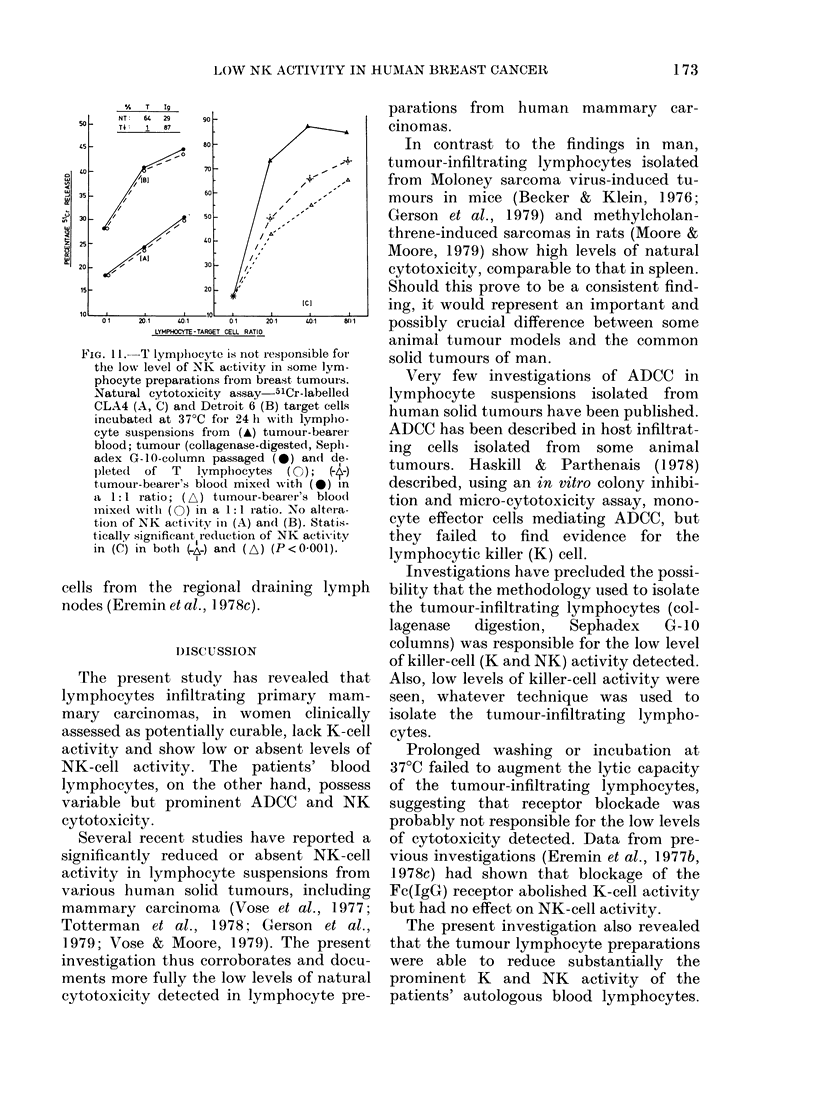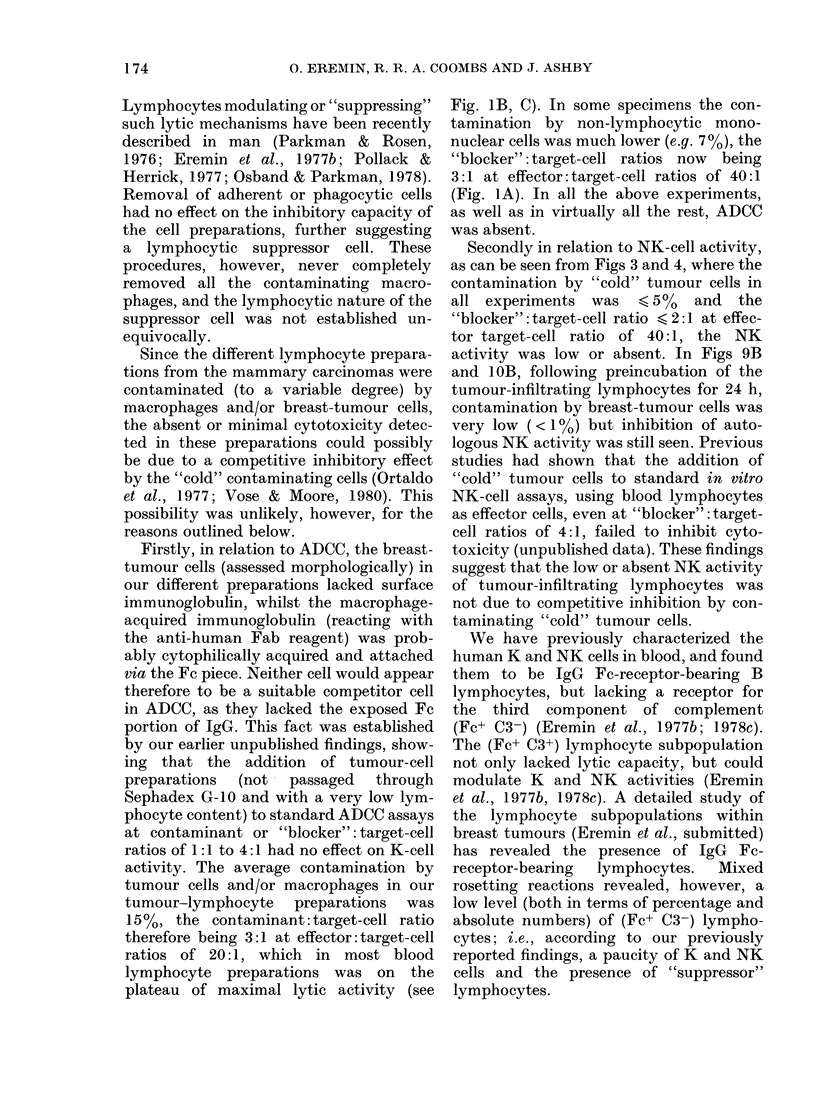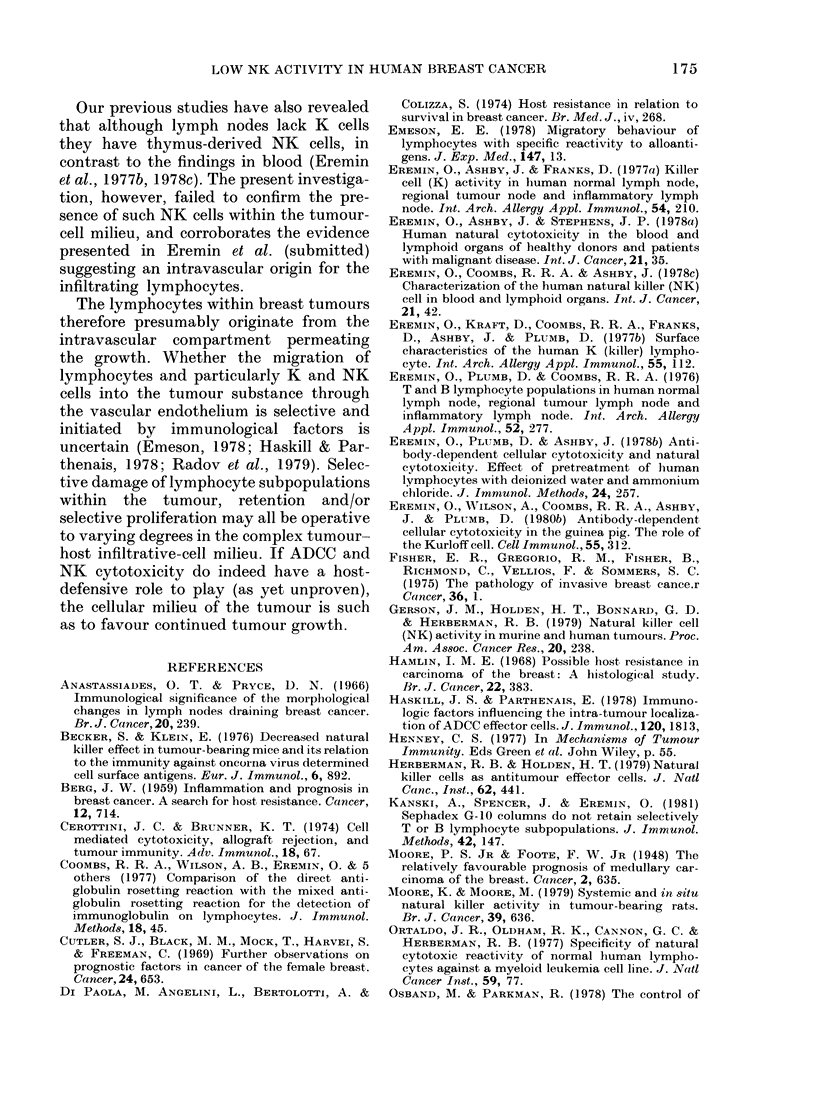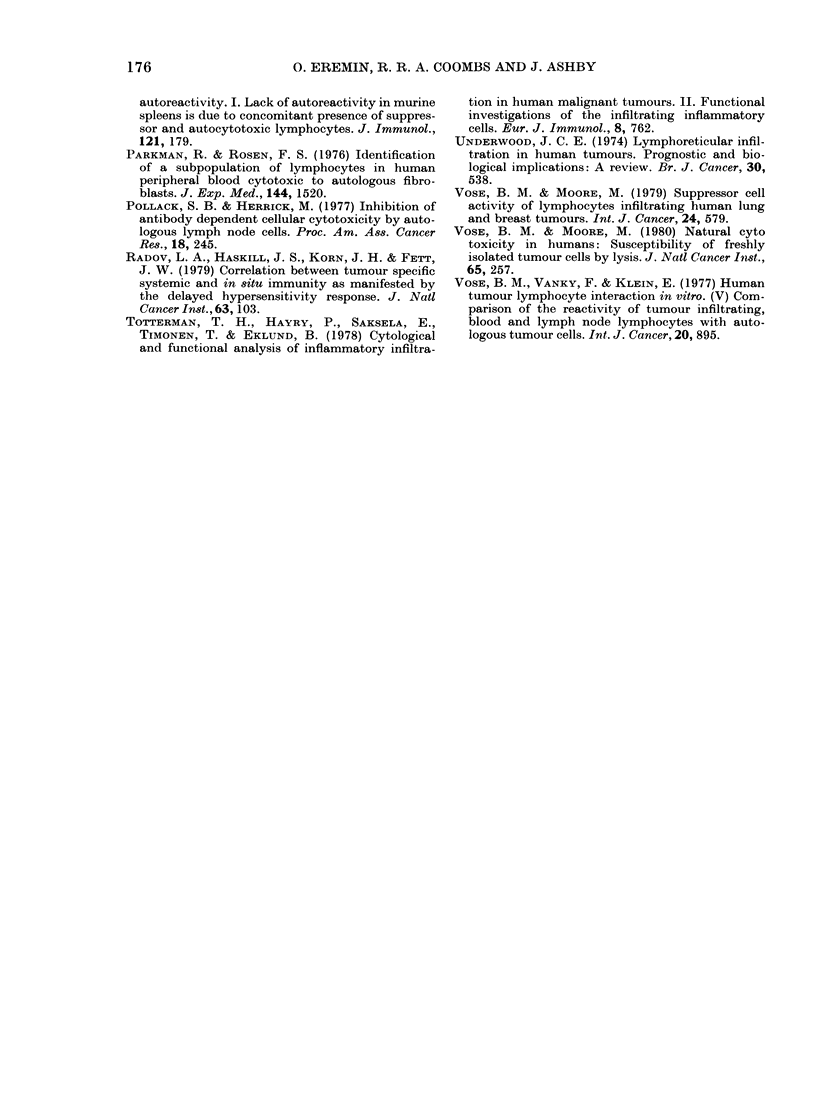# Lymphocytes infiltrating human breast cancers lack K-cell activity and show low levels of NK-cell activity.

**DOI:** 10.1038/bjc.1981.167

**Published:** 1981-08

**Authors:** O. Eremin, R. R. Coombs, J. Ashby

## Abstract

Lymphocytes infiltrating human primary mammary carcinomas lack ADCC and show low levels of natural cytotoxicity. The peripheral blood lymphocytes, however, show a variable but prominent level of cytotoxicity. Lymphocyte preparations from breast tumours, when mixed with autologous blood lymphocytes, significantly suppress their prominent killer- (K- and NK-) cell activities.


Br. J. Cancer (1981) 44, 166

### LYMPHOCYTES INFILTRATING HUMAN BREAST CANCERS LACK K-CELL ACTIVITY AND SHOW LOW LEVELS OF NK-CELL ACTIVITY

O. EREMIN*, R. R. A. COOMBS AND J. ASHBY

*From the Division of Immunology, Department of Pathology, University of Cambridge, Addenbrooke's Hospital, Cambridge CB2 2QQ*

Received 2 October 1980 Accepted 23 April 1981

**Summary.—Lymphocytes infiltrating human primary mammary carcinomas lack ADCC and show low levels of natural cytotoxicity. The peripheral blood lymphocytes, however, show a variable but prominent level of cytotoxicity. Lymphocyte preparations from breast tumours, when mixed with autologous blood lymphocytes, significantly suppress their prominent killer- (K- and NK-) cell activities.**

VARIOUS ANTI-TUMOUR host-defence mechanisms have been postulated to operate in man, and in particular the possible beneficial role of lymphocytic killer cells has been invoked (Cerottini & Brunner, 1974; Henney, 1977; Herberman & Holden, 1979). A possible explanation for the improved survival and reduced potential to metastasize, seen with extensive lymphocytic infiltrates of primary tumours (for review see Underwood, 1974), is the *in situ* tumour cell damage mediated by the infiltrating killer lymphocytes.

The presence of a prominent lymphocytic infiltrate within mammary carcinomas has been found by many investigators to be a favourable prognostic sign (Moore & Foote, 1948; Berg, 1959; Anastassiades & Pryce, 1966; Hamlin, 1968; Cutler *et al.*, 1969; Di Paola *et al.*, 1974; Fisher *et al.*, 1975). The precise anti-tumour role of the tumour-infiltrating lymphocytes, however, is as yet improperly understood.

We have previously described antibody-dependent cellular-cytotoxicity (ADCC) mediated by killer (K) cells, and natural cytotoxicity mediated by natural killer (NK) cells, in the peripheral blood and regional tumour-draining lymph nodes of patients with mammary carcinoma, clinically localized to the breast and axilla (Eremin *et al.*, 1977*a*; 1978*a*). In this communication we report our findings on the K and NK activity of lymphocytes isolated from the primary mammary carcinomas removed at operation from such patients.

### METHODS

### *Blood lymphocyte preparation*

Venous blood, from healthy adult volunteers and patients with breast cancer, was collected by venepuncture into syringes containing preservative-free heparin (30 i.u./ml). The patients were women, aged 40–67 years, with a clinical Stage I or II mammary carcinoma confined to the breast and axilla. Lymphocytes were isolated from the heaprinized blood on a Ficoll–Hypaque gradient, washed and made up in tissue-culture medium (TCM). TCM consisted of RPMI 1640 with 10% heat-inactivated foetal calf serum, (0·7 g/l) streptomycin (100 μg/l) and penicillin G (100,000 i.u./l).

To assess the effect of the various methodological procedures on K and NK activity, some of the blood lymphocytes were further treated as follows: (a) $10^7$ lymphocytes were incubated with 15 ml of collagenase (Sigma,

* Present address: University Department of Clinical Surgery, The Royal Infirmary, Edinburgh EH3 9YW

Type 1—see below) at 37°C for varying periods of time ($\frac{1}{2}$–24 h). (b) $3 \times 10^7$ lymphocytes were passaged down Sephadex G-10 columns (see below) and the emerging cells collected from the eluate. After both treatments the cells were washed ×3 in TCM, counted and viability reassessed.

*Tumour-infiltrating lymphocyte preparation*

*Mechanical disaggregation.*—The breast-tumour specimen was obtained in a sterile manner at operation from the excisional biopsy sample sent for frozen section. The tumour specimen was cleared of fat and fascia and washed in TCM. The specimen was then carefully sliced (Size 10 scalpel blades) into small, thin slices, in a small pot filled with TCM. Spill-out of cells occurred during this procedure. The cell-enriched supernatant was removed, filtered through sterile gauze layers to remove residual stromal fragments and then washed ×5 in TCM. This technique usually produced a low yield of viable cells and a substantial number of necrotic cells which often had to be removed by layering on a Ficoll–Hypaque gradient (sp. gr. 1·077) and centrifuging at 400 *g* for 15 min.

*Enzymatic digestion.*—The small, chopped-up pieces were then incubated in collagenase (Sigma, Type 1) at 37°C for 11–14 h. (The collagenase, 300 u./ml, was dissolved in TCM (protein-free), filter-sterilized and stored at −30°C.) The prolonged incubation of the tumour pieces produced a thick cell suspension, which was washed ×6 with TCM and passed through sterile gauze layers to remove residual stromal fragments and debris. This procedure usually yielded a substantial number of viable cells ($5–50 \times 10^6$) depending primarily on the size of the tumour specimen processed. The lymphocytes, consisting of a variable percentage of the total cell yield, were isolated from such enzyme preparations by passage on Sephadex G-10 columns (see below). Lymphocyte numbers, viability and contamination by macrophages was reassessed in the column eluate. Occasionally there was a heavy contamination by red blood cells, which were removed by lysis with deionized water—a procedure known not to reduce killer-cell activity (Eremin *et al.*, 1978*b*).

Cell size and viability were assessed by phase-contrast microscopy. Surface-marker characteristics were determined by various resetting assays and the data presented in Eremin *et al.* (submitted). Where possible, morphology was characterized further by stained smears or cytocentrifuge preparations.

*Sephadex G-*10 *columns*

Sephadex G-10 columns, as described previously (Eremin *et al.*, 1980*b*; Kanski *et al.*, 1981) were used to isolate lymphocytes from enzymatically digested tumour-cell preparations, the much larger macrophages and tumour cells being trapped on the column. This procedure has been found not to deplete selectively different lymphocyte subsets, irrespective of the source of the lymphocytes.

*Cytotoxicity assays*

ADCC was determined by an *in vitro* 3h, $^{51}$Cr-release assay as previously described (Eremin *et al.*, 1977*a*). The target cells used were CLA4, a lymphoblastoid cell line growing as a suspension culture (Epstein–Barr-virus positive) and Detroit 6 (D6), growing as a monolayer (Eremin *et al.*, 1977*a*). The target cells were coated with human anti-HLA serum (1:500) or with rat IgG anti-D6 antibody (1:400). Effector cells were blood lymphocytes (normal, collagenase-treated and Sephadex G-10 passaged), total-cell preparations from breast tumours (mechanical disaggregation and collagenase digestion), lymphocytes isolated from breast-tumour preparations (collagenase digestion and Sephadex G-10 passage, mechanical disaggregation) and lymphocytes isolated from breast-tumour preparations (collagenase digestion and Sephadex G-10 passage) and depleted of T lymphocytes. The effector:target-cell ratio was 40:1, 20:1 and 0:1.

Natural cytotoxicity was determined by an *in vitro*, 24h, $^{51}$Cr-release assay as described by Eremin *et al.* (1978*a*). The target cells were CLA4 and D6, and the effector cells were as outlined above.

Percentage isotope released was calculated, and assays were statistically evaluated by analysis of variance on the logarithm of the percentage isotope released. Duncan's multiple-range testing was used to assess the significance of the various treatments on ADCC and NK cytotoxicity.

*Lymphocyte surface markers*

Lymphocyte surface markers were determined by various rosetting assays. The thymus-derived E-rosetting lymphocytes were enumerated as described by Eremin *et al.*

(1976). Briefly, sheep red blood cells (1% suspension) were rosetted with lymphocytes ($2 \times 10^6$/ml) in the presence of 30% foetal calf serum (absorbed with SRBC), by centrifuging at 200 *g* for 3 min and allowing to stand for 1 h at room temperature. The sIg-bearing B lymphocytes were detected by the direct antiglobulin rosetting (DAR) assay, using chromic chloride to couple the rabbit anti-human Fab to trypsin-treated ox red blood cells (Coombs *et al.*, 1977).

Surface-marker profiles were done on the following preparations of tumour-infiltrating lymphocytes: (1) lymphocytes isolated by collagenase digestion and passage down Sephadex G-10 columns; and (2) lymphocytes isolated by collagenase digestion, passaged down Sephadex G-10 columns and depleted of E-rosetting T lymphocytes.

*T-lymphocyte depletion*

Tumour-infiltrating lymphocytes, isolated by collagenase digestion and passage down a Sephadex G-10 column, were rosetted in bulk with SRBC (Eremin *et al.*, 1976). The cell mixture (rosetted T lymphocytes, non-rosetted lymphocytes and SRBC) was layered on to a Ficoll–Hypaque gradient (sp. gr. 1·077) and spun with an interface force of 400 *g* at 20°C for 15 min. After centrifugation, the interface band of lymphocytes (free of contaminating SRBC) was removed, washed in TCM and lymphocyte numbers and viability reassessed. The efficacy of the depletion was determined by rosetting. The untreated and T-lymphocyte-depleted preparations were set up in the 24h NK assay.

### RESULTS

*Blood lymphocyte preparations*

*Untreated.*—Lymphocytes isolated from the blood of patients with Stage I or II mammary carcinoma (localized to the breast and axilla) and from healthy controls were 98% viable (phase contrast) and free of contaminating phagocytic cells (< 1% polymorphs, < 3% monocytes).

*Treated.*—(a) Incubation of blood lymphocytes at 37°C with collagenase for short periods (1–4 h) caused minimal cell losses, but incubation for long periods (16–24 h) led to substantial cell losses (30–50%). The final cell suspensions, however, were composed of viable lymphocytes (95%). (b) Passage of lymphocyte suspensions down Sephadex G-10 columns yielded a high percentage of viable cells (> 80% recovery).

*Tumour-infiltrating lymphocyte preparations*

*Mechanical disaggregation.*—A variable but usually low yield of viable cells (macrophages, tumour cells and lymphocytes) was obtained by this technique. Usually most of the cells were necrotic and a further purification procedure, on a Ficoll–Hypaque gradient, was then carried out. Cell suspensions used in the killing assays were either a mixture of lymphocytes (~50%) and other cell types (macrophages and tumour cells) or predominantly lymphocytes (90%) as assessed morphologically.

*Enzymatic digestion.*—Incubation with collagenase yielded a substantially larger number of viable cells (macrophages, tumour cells and lymphocytes) from the tumour specimens ($5–50 \times 10^6$). The macrophages (10–60% of the non-lymphocytic cells) expressed receptors for Fc(IgG) and possessed surface immunoglobulin—presumably acquired cytophilically. The tumour cells (40–90% of the non-lymphocytic cells) lacked receptors for Fc(IgG) and were not coated with immunoglobulin. The latter could not be detected even with the very sensitive DAR assay (Coomb *et al.*, 1977). The tumour-infiltrating lymphocytes (10–50% of the total mononuclear cell population, and in absolute terms $5 \times 10^5–22 \times 10^6$) were composed of both T and B lymphocytes and their various subsets (Eremin *et al.*, submitted). In most cases the tumour-infiltrating lymphocytes were isolated from the tumour-cell preparations by passage through Sephadex G-10 columns. On average, 44% (30–57%) of the tumour-infiltrating lymphocytes were recovered from the column, and on average 85% (76–94%) of the mononuclear cells eluted from the column were lymphocytes, the contaminants being ≥ 70% macrophages.

Most of the breast tumours were scirrous and relatively avascular and the very low erythrocyte:lymphocyte ratio suggested minimal contamination by blood elements.

*Tumour-infiltrating lymphocyte preparations lack K-cell activity*

Figs 1, 2 & 5 show the total absence of ADCC in lymphocyte preparations obtained from primary mammary carcinomas, which was seen whether target cells were coated with human or rat IgG antibodies. This total lack of K-cell activity was seen in 14/16 breast-tumour specimens (Fig. 5). In the remaining 2 specimens there was a very low K-cell activity, the breast-tumour preparation (before column passage) being associated with a relatively heavy contamination by RBC and presumably by K cells, from the intravascular compartment of the tumour. Figs 1 & 2 show that enzymatically obtained tumour-cell preparations (total-cell suspensions from breast tumours), even when not passaged down the Sephadex G-10 column and containing both Fe (IgG)-receptor-bearing macrophages and lymphocytes, similarly lacked ADCC (4 specimens). Tumour-infiltrating lymphocytes, obtained by mechanical means (4 specimens), with or without Sephadex G-10 column passage, also lacked K-cell activity (Figs 1, 2 & 5). Prolonged washing of the lymphocytes (up to 12×) failed to restore the K-cell activity (data not shown); pre-incubating the lymphocytes at 37°C for 24 h also failed to restore the absent ADCC (see Fig. 8). Longer incubations (48–72 h) also failed to restore the ADCC (data not shown).

FIG. 1.—Tumour-infiltrating lymphocytes lack K-cell activity when compared with blood lymphocytes. ADCC assay: $^{51}$Cr-labelled CLA4 target cells, coated with human anti-HLA serum (1:500), incubated at 37°C for 3 h with lymphocytes from: (●) control blood; (▲) tumour-bearer blood; (◆) tumour (collagenase digested); (△) tumour (collagenase-digested, SG-10-column-passaged); (□) tumour (mechanical disaggregation). A, B, C refer to experiments with 3 different patients.

FIG. 2.—As Fig. 1 but with different target cells: $^{51}$Cr-labelled D6 cells, coated with rat IgG anti-Det6 antibody (1:400), incubated at 37°C for 3 h with the lymphocyte suspensions described in Fig. 1.

Peripheral-blood lymphocyte preparations, on the other hand, from all the patients tested showed a variable but prominent K-cell activity (Figs 1, 2 & 5).

*Tumour-infiltrating lymphocyte preparations show a low and significantly reduced NK activity*

The natural cytotoxicity of lymphocytes isolated from primary breast tumours was either low or totally lacking (Figs 3, 4 & 5). This pattern of NK activity was seen (a) whether the method used to prepare

FIG. 3.—Tumour-infiltrating lymphocytes show a very low NK-cell activity against CLA4 target cells as compared with blood lymphocytes. Natural cytotoxicity assay—$^{51}$Cr-labelled CLA4 target cells incubated at 37°C for 24 h with lymphocyte suspensions prepared as described in Fig. 1. (Symbols as in Fig. 1.)

FIG. 4.—Tumour-infiltrating lymphocytes show a low NK-cell activity against D6 target cells as compared with blood lymphocytes. As in Fig. 3, using D6 target cells.

FIG. 5.—Diminished killer-cell (K and NK) activity of 15 tumour-infiltrating lymphoid-cell preparations. ADCC assay—$^{51}$Cr-labelled CLA4 target cells, coated with anti-HLA serum (1:500), incubated at 37°C for 3 h with lymphocytes from the patients' blood and tumour.

Natural cytotoxicity assay—$^{51}$Cr-labelled CLA4 target cells incubated at 37°C for 24 h with lymphocytes from the patients' blood and tumour.

Only maximal lymphocyte: target cell ratio (40:1) shown. The percentage $^{51}$Cr released was estimated by subtracting the background release (targets alone) from the maximal release.

and isolate the lymphocytes was mechanical disaggregation or collagenase digestion (Figs 3 & 4), (b) against both CLA4 and D6 target cells (Figs 3 & 4) and (c) in all the 15 patients investigated (Fig. 5). As in the case of the K cell, prolonged washing (up to 12×), or pre-incubation at 37°C before setting up the 24h *in vitro* assay, failed to raise this low level of natural cytotoxicity (Figs 9 & 10).

The peripheral-blood lymphocytes, on the other hand, from all 15 patients showed a variable but very prominent NK activity (Fig. 5).

*Incubation of blood lymphocytes with collagenase did not reduce K and NK activity*

As can be seen from Fig. 6, pre-incubation of blood lymphocytes with collagenase at 37°C for 4 h and 16 h did not reduce ADCC nor NK cytotoxicity. Incubating for more than 16 h (*e.g.* 24 h) on the other hand did cause significant loss of lymphocytes, low cell viability and NK activities (data not shown). All the breast-tumour specimens were incubated with collagenase for less than 16 h, and the cells from the tumour digest for an even shorter period.

FIG. 6.—Pre-incubation of blood lymphocytes with collagenase does not reduce ADCC or natural cytotoxicity. ADCC assay—$^{51}$Cr-labelled CLA4 target cells, coated with human anti-HLA serum (1:500), incubated at 37°C for 3 h with blood lymphocytes pre-incubated at 37°C without collagenase for 4 h (●), 16 h (▲), with collagenase for 4 h (○), 16 h (△) (see Methods for details).

Natural cytotoxicity assay—$^{51}$Cr-labelled CLA4 target cells incubated at 37°C for 24 h with blood lymphocptes pre-incubated at 37°C without collagenase for 4 h (●), 16 h (▲); with collagenase for 4 h (○), 16 h (△). Enzyme treatment produces no significant difference in ADCC and natural cytotoxicity

FIG. 7.—Passage of blood lymphocytes down Sephadex G-10 columns does not reduce ADCC or natural cytotoxicity. ADCC assay —$^{51}$Cr-labelled CLA4 (A) target cells coated with human anti-HLA serum (1: 500) and $^{51}$Cr-labelled Det6 (B) target cells coated with rat IgG anti-D6 antibody (1: 400) were incubated at 37°C for 3 h with blood lymphocytes prepared in the standard manner (●, ▲)s standard manner and G-10 column passaged (○, △) (see Methods for details).

Natural cytotoxicity assay—$^{51}$Cr-labelled CLA4 (A) and D6 (B) target cells were incubated at 37°C for 24 h with blood lymphocytes prepared in the standard manner (●, ▲); standard manner and G-10-column passaged (○, △).

G-10-column-passage produced no significant difference in ADCC and natural cytotoxicity.

*Passage of blood lymphocytes through Sephadex G-10 columns did not remove K and NK cells*

To exclude the possibility that the absence of K and the low NK activity of the breast-tumour-infiltrating lymphocytes was due to the selective retention of K and NK cells on the Sephadex G-10 column, the ADCC and NK cytotoxicity of blood lymphocytes before and after column passage were determined. The results (Fig. 7) show convincingly that the Sephadex G-10 column did not selectively remove K and NK cells from the blood-lymphocyte suspensions. Sephadex G-10 columns had been shown in a previous study not to deplete different lymphocyte subsets (Kanski *et al.*, 1981).

*Suppression of blood K and NK activities by lymphocyte preparations from breast tumours*

Figs 8, 9 & 10 reveal convincingly that ADCC and NK cytotoxicity of blood lymphocytes could be substantially reduced by the addition of tumour-infiltrating lymphocyte preparations. Previous studies (Eremin *et al.*, 1977*b*; 1978*c*) have shown that the reduction of blood cytotoxicity *in vitro* by the addition of certain lymphocyte subpopulations was not due to nonspecific factors (*e.g.* overcrowding) but to the presence of suppressor or competitive inhibitor cells. In the present study, the latter cells were not removed by pre-

FIG. 8.—Lymphocyte preparations from breast tumours can suppress autologous-blood ADCC. ADCC assay—$^{51}Cr$-labelled CLA4 target cells coated with human anti-HLA antiserum (1:500), incubated at 37°C for 3 h with lymphocytes from [(●) tumour-bearer blood (○) tumour (collagenase-digested—see Methods); (⊙) Mixture of ● and ○ in 1:1 ratio; the lymphocyte: target cell ratio here being 80:1, 40:1 and 0:1. (A) Standard assay. (B) Pre-incubation of lymphocytes. 37°C for 24 h before the standard assay.

Statistically significant reduction of ADCC in (A) and (B) when tumour-infiltrating lymphocyte preparations were added to patients' blood-lymphoctye suspensions ($P < 0{\cdot}001$).

FIG. 9.—Lymphocyte preparations from breast tumours suppress autologous-blood NK activity. Natural cytotoxicity assay—$^{51}Cr$-labelled CLA4 target cells incubated at 37°C for 25 h with lymphocyte suspensions prepared as described in Fig. 8.

Statistically significant reduction of natural cytotoxicity occurred in (A) and (B) when tumour-infiltrating lymphocyte preparations were added to patients' blood-lymphocyte suspensions ($P < 0{\cdot}001$).

incubation of the lymphocyte preparations at 37°C on a glass surface in the presence of 10% foetal calf serum (Figs 8, 9 & 10). Pretreatment of tumour-infiltrating lymphocytes with carbonyl iron was also unsuccessful (data not shown). The lack of glass adherence and phagocytosis suggested a non-macrophage-like cell. We have preliminary data, however, showing reduced phagocytic capacity and poor glass-adherence properties of the macrophages within mammary cancers. Thus, since all the lymphocyte preparations had a small population of contaminating tumour macrophages, it was not possible to determine unequivocally the precise nature of this "suppressor" cell.

*Removal of E-rosetting T lymphocytes fails to alter the low level of NK-cell activity*

The low level of NK activity in some preparations of tumour-infiltrating lym-

FIG. 10.—As in Fig. 9, using D6 target cells.

phocytes was unaltered by depletion of the E-rosetting thymus-derived lymphocytes (Fig. 11). The T depleted population was also able to suppress the prominent NK activity of the patients' blood lymphocytes. The low levels of cytotoxicity, therefore, are unlikely to be due to migration of E-rosetting thymus-derived NK

FIG. 11.—T lymphocyte is not responsible for the low level of NK activity in some lymphocyte preparations from breast tumours. Natural cytotoxicity assay—$^{51}$Cr-labelled CLA4 (A, C) and Detroit 6 (B) target cells incubated at 37°C for 24 h with lymphocyte suspensions from (▲) tumour-bearer blood; tumour (collagenase-digested, Sephadex G-10-column passaged (●) and depleted of T lymphocytes (○); (-△-) tumour-bearer's blood mixed with (●) in a 1:1 ratio; (△) tumour-bearer's blood mixed with (○) in a 1:1 ratio. No alteration of NK activity in (A) and (B). Statistically significant reduction of NK activity in (C) in both (-△-) and (△) ($P < 0.001$).

cells from the regional draining lymph nodes (Eremin *et al.*, 1978*c*).

### DISCUSSION

The present study has revealed that lymphocytes infiltrating primary mammary carcinomas, in women clinically assessed as potentially curable, lack K-cell activity and show low or absent levels of NK-cell activity. The patients' blood lymphocytes, on the other hand, possess variable but prominent ADCC and NK cytotoxicity.

Several recent studies have reported a significantly reduced or absent NK-cell activity in lymphocyte suspensions from various human solid tumours, including mammary carcinoma (Vose *et al.*, 1977; Totterman *et al.*, 1978; Gerson *et al.*, 1979; Vose & Moore, 1979). The present investigation thus corroborates and documents more fully the low levels of natural cytotoxicity detected in lymphocyte preparations from human mammary carcinomas.

In contrast to the findings in man, tumour-infiltrating lymphocytes isolated from Moloney sarcoma virus-induced tumours in mice (Becker & Klein, 1976; Gerson *et al.*, 1979) and methylcholanthrene-induced sarcomas in rats (Moore & Moore, 1979) show high levels of natural cytotoxicity, comparable to that in spleen. Should this prove to be a consistent finding, it would represent an important and possibly crucial difference between some animal tumour models and the common solid tumours of man.

Very few investigations of ADCC in lymphocyte suspensions isolated from human solid tumours have been published. ADCC has been described in host infiltrating cells isolated from some animal tumours. Haskill & Parthenais (1978) described, using an *in vitro* colony inhibition and micro-cytotoxicity assay, monocyte effector cells mediating ADCC, but they failed to find evidence for the lymphocytic killer (K) cell.

Investigations have precluded the possibility that the methodology used to isolate the tumour-infiltrating lymphocytes (collagenase digestion, Sephadex G-10 columns) was responsible for the low level of killer-cell (K and NK) activity detected. Also, low levels of killer-cell activity were seen, whatever technique was used to isolate the tumour-infiltrating lymphocytes.

Prolonged washing or incubation at 37°C failed to augment the lytic capacity of the tumour-infiltrating lymphocytes, suggesting that receptor blockade was probably not responsible for the low levels of cytotoxicity detected. Data from previous investigations (Eremin *et al.*, 1977*b*, 1978*c*) had shown that blockage of the Fc(IgG) receptor abolished K-cell activity but had no effect on NK-cell activity.

The present investigation also revealed that the tumour lymphocyte preparations were able to reduce substantially the prominent K and NK activity of the patients' autologous blood lymphocytes.

Lymphocytes modulating or "suppressing" such lytic mechanisms have been recently described in man (Parkman & Rosen, 1976; Eremin *et al.*, 1977*b*; Pollack & Herrick, 1977; Osband & Parkman, 1978). Removal of adherent or phagocytic cells had no effect on the inhibitory capacity of the cell preparations, further suggesting a lymphocytic suppressor cell. These procedures, however, never completely removed all the contaminating macrophages, and the lymphocytic nature of the suppressor cell was not established unequivocally.

Since the different lymphocyte preparations from the mammary carcinomas were contaminated (to a variable degree) by macrophages and/or breast-tumour cells, the absent or minimal cytotoxicity detected in these preparations could possibly be due to a competitive inhibitory effect by the "cold" contaminating cells (Ortaldo *et al.*, 1977; Vose & Moore, 1980). This possibility was unlikely, however, for the reasons outlined below.

Firstly, in relation to ADCC, the breast-tumour cells (assessed morphologically) in our different preparations lacked surface immunoglobulin, whilst the macrophage-acquired immunoglobulin (reacting with the anti-human Fab reagent) was probably cytophilically acquired and attached *via* the Fc piece. Neither cell would appear therefore to be a suitable competitor cell in ADCC, as they lacked the exposed Fc portion of IgG. This fact was established by our earlier unpublished findings, showing that the addition of tumour-cell preparations (not passaged through Sephadex G-10 and with a very low lymphocyte content) to standard ADCC assays at contaminant or "blocker": target-cell ratios of 1:1 to 4:1 had no effect on K-cell activity. The average contamination by tumour cells and/or macrophages in our tumour–lymphocyte preparations was 15%, the contaminant: target-cell ratio therefore being 3:1 at effector: target-cell ratios of 20:1, which in most blood lymphocyte preparations was on the plateau of maximal lytic activity (see Fig. 1B, C). In some specimens the contamination by non-lymphocytic mononuclear cells was much lower (*e.g.* 7%), the "blocker": target-cell ratios now being 3:1 at effector: target-cell ratios of 40:1 (Fig. 1A). In all the above experiments, as well as in virtually all the rest, ADCC was absent.

Secondly in relation to NK-cell activity, as can be seen from Figs 3 and 4, where the contamination by "cold" tumour cells in all experiments was $\leqslant 5\%$ and the "blocker": target-cell ratio $\leqslant 2:1$ at effector target-cell ratio of 40:1, the NK activity was low or absent. In Figs 9B and 10B, following preincubation of the tumour-infiltrating lymphocytes for 24 h, contamination by breast-tumour cells was very low ($<1\%$) but inhibition of autologous NK activity was still seen. Previous studies had shown that the addition of "cold" tumour cells to standard *in vitro* NK-cell assays, using blood lymphocytes as effector cells, even at "blocker": target-cell ratios of 4:1, failed to inhibit cytotoxicity (unpublished data). These findings suggest that the low or absent NK activity of tumour-infiltrating lymphocytes was not due to competitive inhibition by contaminating "cold" tumour cells.

We have previously characterized the human K and NK cells in blood, and found them to be IgG Fc-receptor-bearing B lymphocytes, but lacking a receptor for the third component of complement ($Fc^+$ $C3^-$) (Eremin *et al.*, 1977*b*; 1978*c*). The ($Fc^+$ $C3^+$) lymphocyte subpopulation not only lacked lytic capacity, but could modulate K and NK activities (Eremin *et al.*, 1977*b*, 1978*c*). A detailed study of the lymphocyte subpopulations within breast tumours (Eremin *et al.*, submitted) has revealed the presence of IgG Fc-receptor-bearing lymphocytes. Mixed rosetting reactions revealed, however, a low level (both in terms of percentage and absolute numbers) of ($Fc^+$ $C3^-$) lymphocytes; *i.e.*, according to our previously reported findings, a paucity of K and NK cells and the presence of "suppressor" lymphocytes.

Our previous studies have also revealed that although lymph nodes lack K cells they have thymus-derived NK cells, in contrast to the findings in blood (Eremin *et al.*, 1977*b*, 1978*c*). The present investigation, however, failed to confirm the presence of such NK cells within the tumour-cell milieu, and corroborates the evidence presented in Eremin *et al.* (submitted) suggesting an intravascular origin for the infiltrating lymphocytes.

The lymphocytes within breast tumours therefore presumably originate from the intravascular compartment permeating the growth. Whether the migration of lymphocytes and particularly K and NK cells into the tumour substance through the vascular endothelium is selective and initiated by immunological factors is uncertain (Emeson, 1978; Haskill & Parthenais, 1978; Radov *et al.*, 1979). Selective damage of lymphocyte subpopulations within the tumour, retention and/or selective proliferation may all be operative to varying degrees in the complex tumour–host infiltrative-cell milieu. If ADCC and NK cytotoxicity do indeed have a host-defensive role to play (as yet unproven), the cellular milieu of the tumour is such as to favour continued tumour growth.

### REFERENCES

Anastassiades, O. T. & Pryce, D. N. (1966) Immunological significance of the morphological changes in lymph nodes draining breast cancer. *Br. J. Cancer*, **20**, 239.

Becker, S. & Klein, E. (1976) Decreased natural killer effect in tumour-bearing mice and its relation to the immunity against oncorna virus determined cell surface antigens. *Eur. J. Immunol.*, **6**, 892.

Berg, J. W. (1959) Inflammation and prognosis in breast cancer. A search for host resistance. *Cancer*, **12**, 714.

Cerottini, J. C. & Brunner, K. T. (1974) Cell mediated cytotoxicity, allograft rejection, and tumour immunity. *Adv. Immunol.*, **18**, 67.

Coombs, R. R. A., Wilson, A. B., Eremin, O. & 5 others (1977) Comparison of the direct antiglobulin rosetting reaction with the mixed antiglobulin rosetting reaction for the detection of immunoglobulin on lymphocytes. *J. Immunol. Methods*, **18**, 45.

Cutler, S. J., Black, M. M., Mock, T., Harvei, S. & Freeman, C. (1969) Further observations on prognostic factors in cancer of the female breast. *Cancer*, **24**, 653.

Di Paola, M. Angelini, L., Bertolotti, A. & Colizza, S. (1974) Host resistance in relation to survival in breast cancer. *Br. Med. J.*, iv, 268.

Emeson, E. E. (1978) Migratory behaviour of lymphocytes with specific reactivity to alloantigens. *J. Exp. Med.*, **147**, 13.

Eremin, O., Ashby, J. & Franks, D. (1977*a*) Killer cell (K) activity in human normal lymph node, regional tumour node and inflammatory lymph node. *Int. Arch. Allergy Appl. Immunol.*, **54**, 210.

Eremin, O., Ashby, J. & Stephens, J. P. (1978*a*) Human natural cytotoxicity in the blood and lymphoid organs of healthy donors and patients with malignant disease. *Int. J. Cancer*, **21**, 35.

Eremin, O., Coombs, R. R. A. & Ashby, J. (1978*c*) Characterization of the human natural killer (NK) cell in blood and lymphoid organs. *Int. J. Cancer*, **21**, 42.

Eremin, O., Kraft, D., Coombs, R. R. A., Franks, D., Ashby, J. & Plumb, D. (1977*b*) Surface characteristics of the human K (killer) lymphocyte. *Int. Arch. Allergy Appl. Immunol.*, **55**, 112.

Eremin, O., Plumb, D. & Coombs, R. R. A. (1976) T and B lymphocyte populations in human normal lymph node, regional tumour lymph node and inflammatory lymph node. *Int. Arch. Allergy Appl. Immunol.*, **52**, 277.

Eremin, O., Plumb, D. & Ashby, J. (1978*b*) Antibody-dependent cellular cytotoxicity and natural cytotoxicity. Effect of pretreatment of human lymphocytes with deionized water and ammonium chloride. *J. Immunol. Methods*, **24**, 257.

Eremin, O., Wilson, A., Coombs, R. R. A., Ashby, J. & Plumb, D. (1980*b*) Antibody-dependent cellular cytotoxicity in the guinea pig. The role of the Kurloff cell. *Cell Immunol.*, **55**, 312.

Fisher, E. R., Gregorio, R. M., Fisher, B., Richmond, C., Vellios, F. & Sommers, S. C. (1975) The pathology of invasive breast cance.r *Cancer*, **36**, 1.

Gerson, J. M., Holden, H. T., Bonnard, G. D. & Herberman, R. B. (1979) Natural killer cell (NK) activity in murine and human tumours. *Proc. Am. Assoc. Cancer Res.*, **20**, 238.

Hamlin, I. M. E. (1968) Possible host resistance in carcinoma of the breast: A histological study. *Br. J. Cancer*, **22**, 383.

Haskill, J. S. & Parthenais, E. (1978) Immunologic factors influencing the intra-tumour localization of ADCC effector cells. *J. Immunol.*, **120**, 1813,

Henney, C. S. (1977) In *Mechanisms of Tumour Immunity*. Eds Green *et al.* John Wiley, p. 55.

Herberman, R. B. & Holden, H. T. (1979) Natural killer cells as antitumour effector cells. *J. Natl Canc., Inst.*, **62**, 441.

Kanski, A., Spencer, J. & Eremin, O. (1981) Sephadex G-10 columns do not retain selectively T or B lymphocyte subpopulations. *J. Immunol. Methods*, **42**, 147.

Moore, P. S. Jr & Foote, F. W. Jr (1948) The relatively favourable prognosis of medullary carcinoma of the breast. *Cancer*, **2**, 635.

Moore, K. & Moore, M. (1979) Systemic and *in situ* natural killer activity in tumour-bearing rats. *Br. J. Cancer*, **39**, 636.

Ortaldo, J. R., Oldham, R. K., Cannon, G. C. & Herberman, R. B. (1977) Specificity of natural cytotoxic reactivity of normal human lymphocytes against a myeloid leukemia cell line. *J. Natl Cancer Inst.*, **59**, 77.

Osband, M. & Parkman, R. (1978) The control of autoreactivity. I. Lack of autoreactivity in murine spleens is due to concomitant presence of suppressor and autocytotoxic lymphocytes. *J. Immunol.*, **121**, 179.

Parkman, R. & Rosen, F. S. (1976) Identification of a subpopulation of lymphocytes in human peripheral blood cytotoxic to autologous fibroblasts. *J. Exp. Med.*, **144**, 1520.

Pollack, S. B. & Herrick, M. (1977) Inhibition of antibody dependent cellular cytotoxicity by autologous lymph node cells. *Proc. Am. Ass. Cancer Res.*, **18**, 245.

Radov, L. A., Haskill, J. S., Korn, J. H. & Fett, J. W. (1979) Correlation between tumour specific systemic and *in situ* immunity as manifested by the delayed hypersensitivity response. *J. Natl Cancer Inst.*, **63**, 103.

Totterman, T. H., Hayry, P., Saksela, E., Timonen, T. & Eklund, B. (1978) Cytological and functional analysis of inflammatory infiltration in human malignant tumours. II. Functional investigations of the infiltrating inflammatory cells. *Eur. J. Immunol.*, **8**, 762.

Underwood, J. C. E. (1974) Lymphoreticular infiltration in human tumours. Prognostic and biological implications: A review. *Br. J. Cancer*, **30**, 538.

Vose, B. M. & Moore, M. (1979) Suppressor cell activity of lymphocytes infiltrating human lung and breast tumours. *Int. J. Cancer*, **24**, 579.

Vose, B. M. & Moore, M. (1980) Natural cyto toxicity in humans: Susceptibility of freshly isolated tumour cells by lysis. *J. Natl Cancer Inst.*, **65**, 257.

Vose, B. M., Vanky, F. & Klein, E. (1977) Human tumour lymphocyte interaction *in vitro*. (V) Comparison of the reactivity of tumour infiltrating, blood and lymph node lymphocytes with autologous tumour cells. *Int. J. Cancer*, **20**, 895.